# Sol-gel synthesis, structure, and dielectric properties of La_0.67_Li_x_Ti_1-x_Al_x_O_3_ solid solutions

**DOI:** 10.1016/j.heliyon.2023.e15392

**Published:** 2023-04-12

**Authors:** Tetiana Plutenko, Oleg V’yunov, Oleksandr Fedorchuk, Borys Khomenko, Anatolii Belous

**Affiliations:** V.I. Vernadsky Institute of General and Inorganic Chemistry of the National Academy of Sciences of Ukraine, Palladina Ave., 32/34, 03142, Kyiv, Ukraine

**Keywords:** Li-containing, Capacitors, Perovskite, Pechini method

## Abstract

La_0.67_Li_x_Ti_1-x_Al_x_O_3_ were synthesized using the sol-gel Pechini route. Ceramic samples were sintered in the temperature range of 1240–1300 °C in the air atmosphere. It was found that in the concentration range of 0.05 ≤ *x* < 0.15 there is a morphotropic phase boundary region with rhombohedral and tetragonal syngonies. In the concentration range of 0.15 ≤ *x* ≤ 0.3, a single-phase solid solution with rhombohedral R $\bar{3}$ c syngony is formed. As the value of x increases, the average grain size of La_0.67_Li_x_Ti_1-x_Al_x_O_3_ ceramics increases from 5.23 μm (*x* = 0.05) to 8.76 μm (*x* = 0.3). All materials of the La_0.67_Li_x_Ti_1-x_Al_x_O_3_ system at 0.05 ≤ *x* ≤ 0.3 have colossal values of dielectric constant ε′ > 10^4^ at frequencies up to 1 kHz. La_0.67_Li_x_Ti_1-x_Al_x_O_3_ (*x* = 0.2) solid solution with rhombohedral syngony has the highest value of dielectric constant and the lowest value of the dielectric losses.

## 1 Introduction

Dielectric materials with colossal dielectric constant with low loss are a rapidly developing research interest in the field of artificially structured metamaterials and offer additional investigations [[1], [2], [3]]. Materials with a high dielectric constant (ε > 1000) are also developed on lithium-containing perovskites and can be used in microelectronics to solve problems of miniaturization of electronic circuits [4]. Materials with a high effective dielectric constant which is provided by the relaxation of mobile lithium ions are of considerable scientific and practical interest. To improve the electrical characteristics, the simultaneous substitution of La and Ti ions by Li and Al ions in the La_2/3_Li_x_Ti_1-x_Al_x_O_3_ system was investigated by the authors of [5,6]. It was found that depending on the sintering conditions La_2/3_Li_x_Ti_1-x_Al_x_O_3_ perovskites can crystallize in orthorhombic or rhombohedral syngony. Material with a high-temperature orthorhombic phase is formed after sintering in the air atmosphere at 1300 °C, followed by quenching. Solid solutions with rhombohedral syngony are formed after sintering in the temperature range 1270–1320 °C with subsequent slow cooling (200°/h). La_2/3_Li_x_Ti_1-x_Al_x_O_3_ materials with orthorhombic syngony form solid solutions in the concentration range *х* = 0.15–0.30 and show a high dielectric constant [6], however, there are no studies of the crystal structure and dielectric properties in the literature in the concentration ranges *x* less than 0.15 and at *x* more than 0.3. For materials with rhombohedral syngony, the regions of the existence of solid solutions have not been determined, and the nature of the influence of substitute ions on the electrical and physical properties has not been clarified.

The conventional method for preparing powders used in ceramic materials production involves roasting a mixture of metal oxides and carbonates in specific proportions [7]. However, the process of grinding the mixture to obtain a finely dispersed state introduces pollutants from abrasive materials that can impair the dielectric properties of the final product. A fully reacted and homogeneous product necessitates a uniform distribution of each substance in the solid-state reaction, which is a diffusion-controlled process. The mechanically ground mixture requires prolonged calcination at high temperatures under precise atmospheric control, which results in the loss of volatile components such as lithium from the solid solution. Additionally, achieving good density requires a very high sintering temperature. To reduce the sintering temperature, a promising approach is the use of a mild, wet chemical technique [8], which produces finer powder morphologies than those obtained by solid-state methods. A complex polymerization process, such as the modified sol-gel Pechini method [9], is an attractive way to obtain oxide powders with high phase purity. In this synthetic procedure, precursor metal ions in the solution are chelated to form metal complexes, which are then polymerized to form a gel.

Therefore, this work aimed to study the crystal structure and dielectric properties of La_0.67_Li_*x*_Ti_1-*x*_Al_*x*_O_3_ solid solutions, which crystallize in rhombohedral syngony synthesized using the sol-gel Pechini method.

## 2 Materials and methods

Stoichiometric amounts of LiNO_3_ (Alfa Assar 99%), La(NO_3_)_3_·6H_2_O (Alfa Assar 99.9%), Ti[OCH(CH_3_)_2_]_4_ (Aldrich 97%), Al(NO_3_)_3_·9H_2_O (Aldrich 98%), (CH_2_)_2_(OH)_2_ (Aldrich 99.8%), and C_6_H_8_O_7_ (Aldrich 99.5%) were used as initial reagents for the synthesis of solid solutions of La_0.67_Li_x_Ti_1-x_Al_x_O_3_ by the sol-gel Pechini method. The total concentration of metal nitrates in the water solution was 0.2 mol/L, with the total weight of metal nitrates ranging from 66.7 to 81.1 g per liter of solution. To prepare the mixture, the nitrate salts of metals (Li, La, Al) and citric acid (CA) were dissolved in distilled water. Meanwhile, titanium isopropoxide Ti[OCH(CH_3_)_2_]_4_ was added to ethylene glycol (EG) under constant stirring. Subsequently, the mixture of citric acid and nitrate salts was added to the titanium solution at a molar ratio of citric acid to metal nitrate of 3:1. The molar ratio of citric acid to ethylene glycol was 1:5, and the amount of titanium isopropoxide was determined based on the product stoichiometry. The homogeneous solution was heated at 100 °C for 12 h to form a resin, which was further heated at 80 °C for 12 h to obtain a dried gel. The dried gel was then calcined at 400 °C for 6 h to obtain the ash, followed by calcination in air for 4 h at 1100 °C to obtain the final product.

The single-phase products were characterized by X-ray powder diffraction using a DRON-4-07 diffractometer (Cu Kα radiation; 40 kV, 20 mA). The unit cell parameters of the samples were determined using FullProf software according to the Le Bail procedure [10]. Powders after heat treatment were ground and compressed into tablets under the pressure of 500 kg/cm^2^ (50 MPa). The materials were sintered in the temperature range of 1240–1300 °C depending on the Li and Al contents, the production of dense ceramics required different sintering temperatures. The tablets were sintered for 6 h and cooled to room temperature at a cooling rate of 200°/h.

The grain sizes of La_0.67_Li_x_Ti_1-x_Al_x_O_3_ (0.05 ≤ *x* ≤ 0.3) ceramic samples were determined using a scanning electron microscope SEC miniSEM SNE 4500 MB equipped with EDAX Element PV6500/00 F spectrometer. Using the imageJ [11] calculations by the method of the equivalent circle diameter were performed [12]. Sampling was performed in three different regions based on at least 50 grains in each region. Sintered cylindrical samples with a diameter of 8 mm and a thickness of 2 mm with metal electrodes were used to measure the dielectric properties. Impedance spectroscopy measurements were conducted using a 1260 Impedance/Gain phase Analyzer (Solartron Analytical).

## 3 Results and discussion

**Fig. 1:**
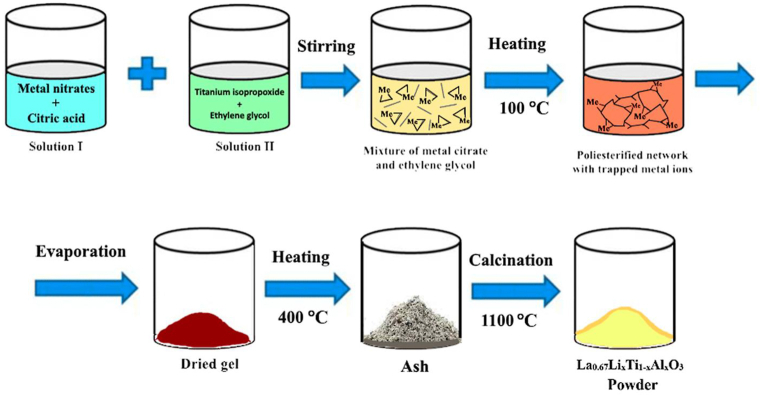
Scheme of the sol-gel Pechini method for synthesis of La_0.67_Li_x_Ti_1-x_Al_x_O_3_. A single-phase La_0.67_Li_x_Ti_1-x_Al_x_O_3_ material with a perovskite structure was synthesized using the sol-gel Pechini method.

Fig. 1 illustrates the synthesis scheme of La_0.67_Li_x_Ti_1-x_Al_x_O_3_ powder using the sol-gel Pechini method. The process involves the formation of a chelate between mixed cations (Li, La, Al, and Ti) and citric acid. The chelates are then cross-linked with ethylene glycol to form a gel through esterification. Two primary chemical reactions take place during the Pechini process for creating La_0.67_Li_x_Ti_1-x_Al_x_O_3_ powder: chelation between complex cations and citric acid, and polyesterification of excess citric acid with ethylene glycol in a slightly acidified solution. The viscous liquid is subsequently dried to obtain a gelatinous precursor for ceramic powders. Final calcination at high temperature eliminates all organic substances, yielding oxide powders of La_0.67_Li_x_Ti_1-x_Al_x_O_3_.

**Fig. 2:**
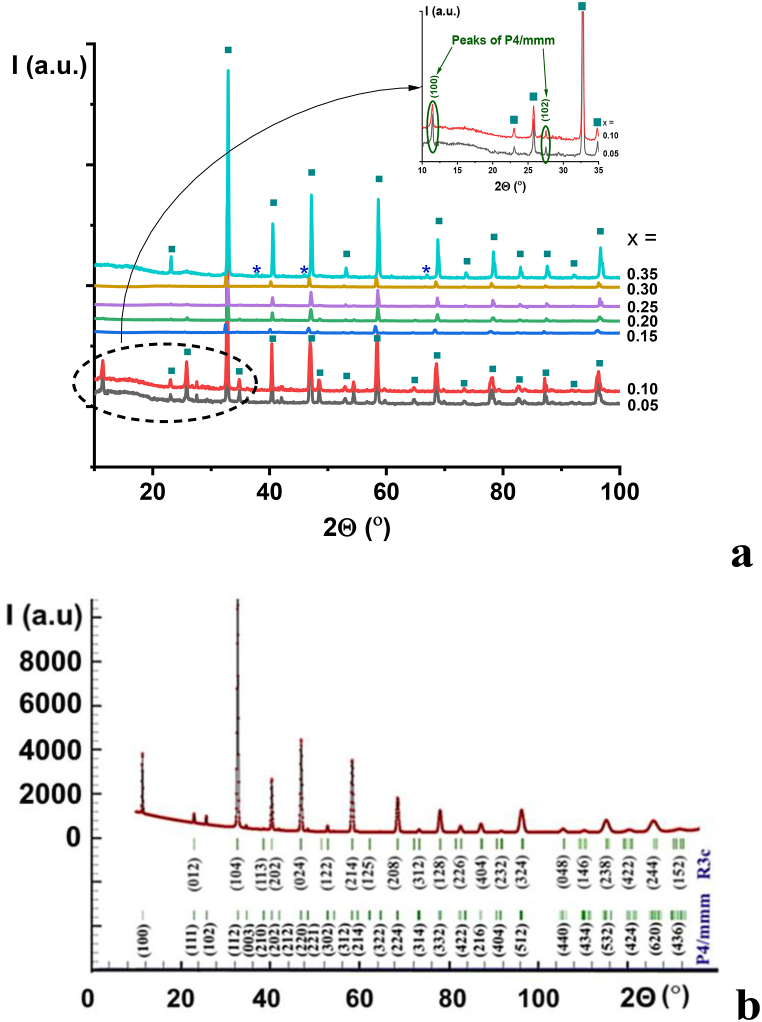
a – XRD patterns of solid solutions of La_0.67_Li_*x*_Ti_1-*x*_Al_*x*_O_3_; b – experimental (dots) and calculated (lines) room-temperature powder X-ray diffraction patterns of La_0.67_Li_0.05_Al_0.05_Ti_0.95_TiO_3_ ceramic sample, sintered at 1300 °C for 2 h. Bars indicate the peak positions.

The material was formed at temperatures above 1100 °C, which is 100° lower compared to the solid-state reaction method [6]. The defect structure of lanthanum titanate La_0.67_TiO_3_ is characterized by tetragonal syngony [13]. Upon the introduction of lithium and aluminum ions into lanthanum titanate into sublattices A and B of perovskite ABO_3_, respectively, a gradual change in the syngony of the solid solution was observed (Fig. 2a). Solid solutions were formed in the concentration range of 0.05 ≤ x ≤ 0.3 in the La_0.67_Li_x_Ti_1-x_Al_x_O_3_ system. At low concentrations of lithium in La_0.67_Li_x_Ti_1-x_Al_x_O_3_ solid solutions, i.e., 0.05 ≤ x < 0.15, the formation of two phases with the same chemical composition but different syngony was observed. The analysis of XRD patterns showed that tetragonal P4/mmm and rhombohedral R $\bar{3}$ c syngonies coexist in the mentioned solid solutions (Fig. 2b).

**Fig. 3:**
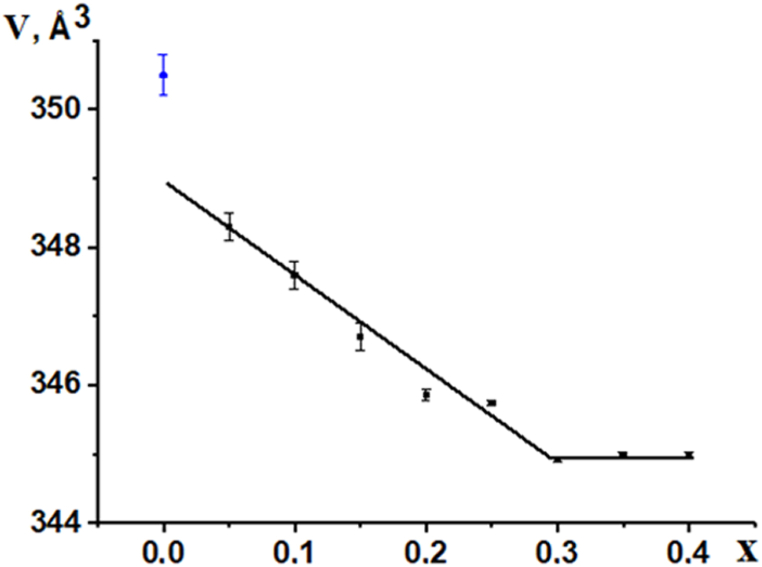
The unit cell volume of La_0.67_Li_*x*_Ti_1-*x*_Al_*x*_O_3_ solid solutions, sintered in the temperature range 1240–1300 °C for 2 h, followed by slow cooling (200°/h).

In La_0.67_Li_*x*_Ti_1-*x*_Al_*x*_O_3_ solid solutions at 0.3 > *x,* there is a non-single-phase region, which is confirmed by the presence of additional peaks at small angles (2Θ < 30°). Upon further replacement of titanium with aluminum (*x* > 0.3), the solid solution decomposes with the formation of additional phases, additional peaks appear on the XRPD patterns, and a broadening of the peaks was observed. In the range of concentrations 0.15 ≤ *x* ≤ 0.3, a single-phase solid solution was formed (Fig. 3), which is characterized by a rhombohedral R $\bar{3}$ c syngony. Fig. 3 shows the calculated unit cell volumes. The volume value for the single-phase material La_0.67_TiO_3_ was taken from the literature [14,15]. The dependence of the unit cell volume in the concentration region 0.05 ≤ *x* ≤ 0.3 is linear and obeys Wegard's law indicating the formation of a continuous series of solid solutions. The unit cell volume with increasing x reduces due to the decrease in the average ionic radius in the titanium sublattice, whereas in the range of concentrations 0.3 < *x* ≤ 0.4, the non-single-phase materials were formed.

**Fig. 4:**
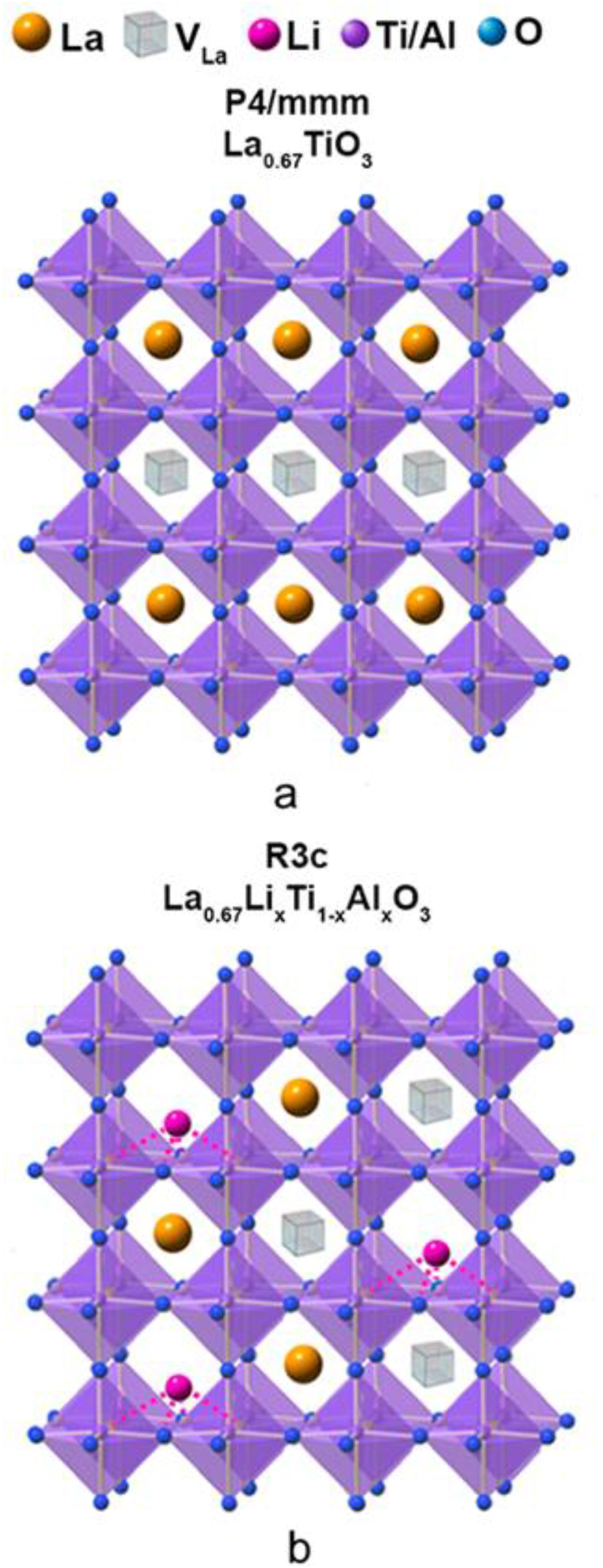
Crystal structure of tetragonal P4/mmm La_0.67_TiO_3_ (a) and rhombohedral R $\bar{3}$ c La_0.67_Li_*x*_Ti_1-*x*_Al_*x*_O_3_ (b).

Yokoyama et al. [15] showed that there is an ordered distribution of lanthanum ions and vacancies in the sublattice A of perovskite-type ABO_3_ (Fig. 4a) in the tetragonal (P4/mmm) lanthanum titanate La_0.67_TiO_3_. In the La_0.67_Li_*x*_Ti_1-*x*_Al_*x*_O_3_ crystallites with rhombohedral R $\bar{3}$ c syngony, a statistical distribution of lanthanum, lithium, and vacancies in the sublattice A is observed (Fig. 4b). It should be noted that lithium ions with a small ionic radius are located not inside the oxygen cuboctahedron, but are shifted in the direction of the plane formed by the four oxygen (Fig. 4b).

**Fig. 5:**
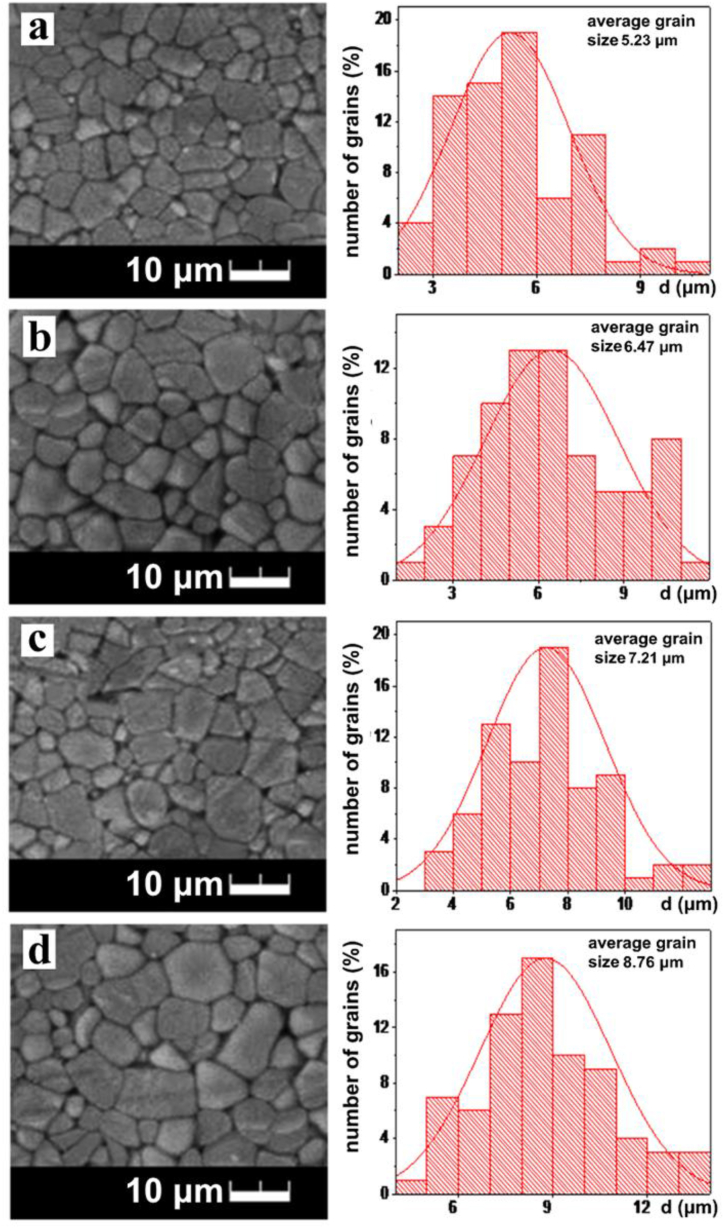
Microstructure of ceramic La_0.67_Li_*x*_Ti_1-*x*_Al_*x*_O_3_ solid solutions, sintered in the temperature range 1240–1300 °C for 2 h followed by slow cooling (200°/h), *x* = 0.05 (a), 0.1 (b), 0.2 (c), 0.3 (d).

Sintering of ceramic materials that are synthesized by sol-gel synthesis occurs by 30–50° less than during synthesis by the solid-state method [6]. The morphology of ceramic samples La_0.67_Li_*x*_Ti_1-*x*_Al_*x*_O_3_ where *x* = 0.05 (Fig. 5a), 0.1 (Figs. 5b), 0.2 (Fig. 5c), 0.3 (Fig. 5d) was shown. With increasing *x*, the average grain size increases from 5.23 μm (*x* = 0.05) to 8.76 μm (*x* = 0.3). This is due to an increase in the sintering temperature of ceramics with increasing content of Li and Al.

During sintering under the influence of temperature, there is a consolidation and strengthening of ceramics, which is accompanied by a decrease in porosity. At the elimination of pores and defects of a lattice at sintering, there is a migration of intergrain borders. As the temperature increases, the boundary between grains gradually decreases, and larger grains are formed.

Lanthanum lithium titanate is a complex oxide material that is known for its interesting electrical properties. It demonstrates colossal dielectric constant and also the grains of the material have a high conductivity, which is attributed to the presence of vacancies in the crystal lattice. These vacancies provide mobile charge Li^+^ ions that can diffuse through the crystal lattice of lanthanum lithium titanates, resulting in high overall ionic conductivity of the material [5,6,16].

**Fig. 6:**
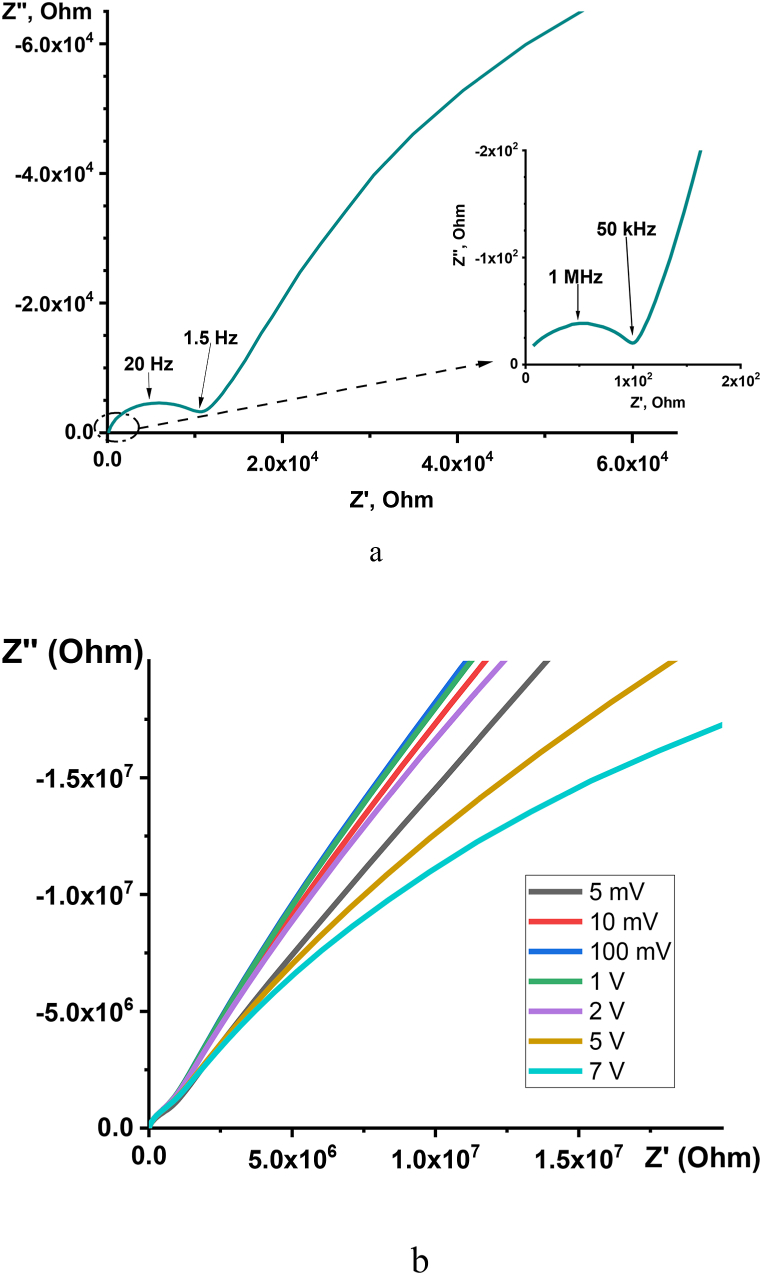
Complex impedance diagram of La_0.67_Li_0.2_Ti_0.8_Al_0.2_O_3_ at room temperature on 100 mV (a). The impedance of La_0.67_Li_0.2_Ti_0.8_Al_0.2_O_3_ is measured with different voltages (b).

Fig. 6a shows that there are two semicircles and a straight line in the Cole-Cole plots of La_0.67_Li_x_Ti_1-x_Al_x_O_3_ materials. Therefore, there are three main polarization mechanisms overall and two relaxation mechanisms, particularly in the frequency range of 10^−2^ to 10^7^ Hz. The straight line is part of a large semicircle (Fig. 6b) and may be assigned to the electrode polarization and described by the Warburg element model with the semi-infinite diffusion layer [17]. The nature of relaxation mechanisms can be revealed by studying the dielectric properties of materials.

**Fig. 7:**
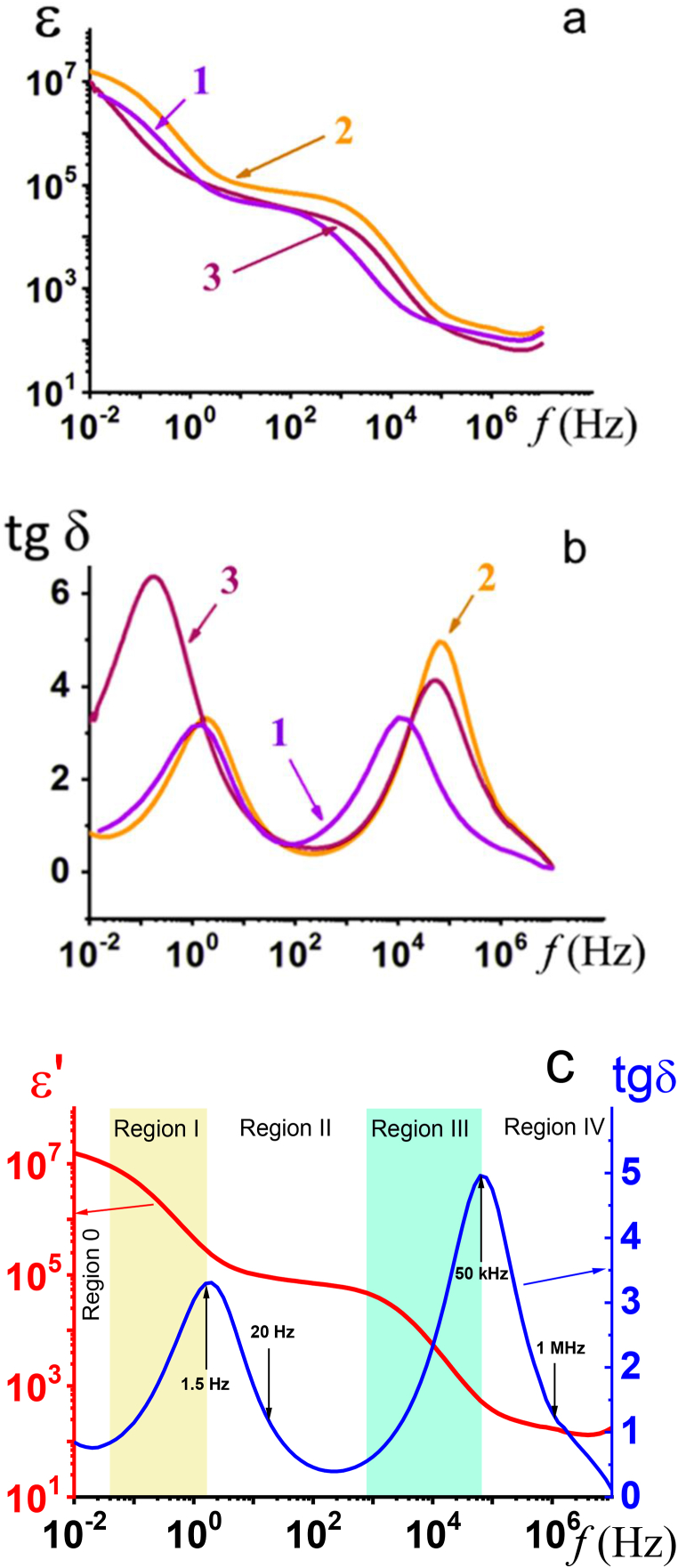
Dielectric constant (a), dielectric loss tangent (b) of La_0.67_Li_*x*_Ti_1-*x*_Al_*x*_O_3_ solid solutions, sintered in the temperature range 1240–1300 °C for 2 h followed by slow cooling (200°/h) at *x* = 0.05 (1), 0.2 (2), 0.3 (3). The dielectric constant and dielectric loss tangent of La_0.67_Li_0.2_Ti_0.8_Al_0.2_O_3_ solid solution.

The frequency dependences of the dielectric constant and the dielectric loss tangent for the La_0.67_Li_*x*_Ti_1-*x*_Al_*x*_O_3_ are shown in Fig. 7a and b. All samples of the La_0.67_Li_*x*_Ti_1-*x*_Al_*x*_O_3_ system at 0.05 ≤ *x* ≤ 0.3 have high values of the dielectric constant ε′ > 10^4^ in the frequency range of 0.01 ≤ *f* ≤ 10^3^ Hz. Solid solution La_0.67_Li_*x*_Ti_1-*x*_Al_*x*_O_3_, where *x* = 0.2, with rhombohedral syngony, was characterized by the highest value of the dielectric constant and the lowest value of the dielectric loss tangent (Fig. 7b). The dependences demonstrate 5 characteristic regions of dielectric constant and loss tangent (Fig. 7c).

The region I (frequency range from 0.03 Hz to ∼ 2 Hz) corresponds to the low-frequency Cole-Cole relaxation semicircle. Such relaxation frequencies are characteristic of migratory polarization [18]. The migratory polarization is provided by the presence of a large number of mobile lithium ions. Lithium ions move over long distances, up to several grain sizes. They overcome a potential barrier at grain boundaries to move between positions in different elementary cells. Migratory polarization turns off completely at the frequency of around 1–2 Hz, which is confirmed by the peak on the loss tangent curve.

The second high-frequency semicircle in Cole–Cole plots is associated with relaxation at frequencies around 10^3^–10^5^ Hz, the region III in Fig. 7. It may be caused by the ion relaxation polarization [19]. Ion-relaxation polarization is associated with the movement of Li^+^ ions within the unit cell. In this case, there is no potential barrier to their movement, and the dielectric constant of the material remains constant when an electric field is applied. And it also relaxes in region III from 10^3^ Hz to 10^5^ Hz (the second peak on the tangent loss curve).

The elastic ion displacement polarization is dominant in the region from 10^5^ Hz (region IV, Fig. 7c).

It is known that in perovskite La_0.67_Li_*x*_Ti_1-*x*_Al_*x*_O_3_ solid solutions lanthanum ions have a coordination number of 12 and occupy a position in the center of the cuboctahedron (Fig. 4b). The lithium ions in the La_0.67_Li_*x*_Ti_1-*x*_Al_*x*_O_3_ perovskite structure are characterized by a coordination number of 4 and have square-plane coordination. Lithium ions are not located in the center of the cuboctahedron but are shifted in the direction of four oxygen (Fig. 4b). It should be noted that the movement of lithium ions can promote both ionic conductivity and polarization. To increase the polarizability of the material, it is necessary to limit the movement of lithium ions in structural channels by the introduction of ions with a large ionic radius, such as lanthanum ions. The dielectric constant depends on the number of charge carriers involved in relaxation polarization and the distance at which they can be displaced. The high values of the dielectric constant in La_0.67_Li_0.2_Ti_0.8_Al_0.2_O_3_ can be explained by the significant amount of lithium ions involved in relaxation, vacancies, and favorable steric conditions. As the lithium concentration increases to *x* ≥ 0.2, the volume of the unit cell decreases, which leads to a decrease in the lithium ions' mobility and, accordingly, to a reduction in the dielectric constant.

The obtained La_0.67_Li_x_Ti_1-x_Al_x_O_3_ materials were compared with La_0.5_Li_0.5-x_Na_x_TiO_3_ materials. It was shown that the materials in this article synthesized by sol-gel method with the addition of aluminum La_0.67_Li_0.2_Ti_0.8_Al_0.2_O_3_ exhibit the value of the dielectric constant of 458000–5570 in the frequency range of 1–10^4^ Hz (tan δ_min_ = 0.36) while materials based on La_0.5_Li_0.5_TiO_3_ show a dielectric constant of 8600–800 (1–10^4^ Hz, tan δ_min_ = 0.25) [20]. The materials of the La_0.5_Li_0.4_Na_0.1_TiO_3_ system exhibit dielectric constant values in the range of 40100–12000 (1–10^4^ Hz, tan δ_min_ = 0.39) [20]. Therefore, it was shown that La_0.67_Li_x_Ti_1-x_Al_x_O_3_ materials containing aluminum exhibit higher values of dielectric constant at the wide frequency range compared with other La_0.5_Li_0.5-x_Na_x_TiO_3_-based materials.

## 4 Conclusions

In this work, novel Li-conducting solid solutions La_0.67_Li_*x*_Ti_1-*x*_Al_*x*_O_3_ with rhombohedral structure were prepared by the sol-gel Pechini technique. It was shown that the use of the sol-gel Pechini synthesis method makes it possible to reduce the calcination temperature of ceramics by 100° and the sintering temperature by 30–50°, depending on the concentration *x*, in comparison with the solid-state reaction technique. The influence of aluminum and lithium on the crystal lattice parameters in a wide concentration range was shown. A single-phase solid solution with rhombohedral syngony exists in a concentration range of 0.15 ≤ *x* ≤ 0.3. In the concentration range of 0.05 ≤ *x* < 0.15, the coexistence of rhombohedral and tetragonal syngonies was established. The volume of the unit cell decreases with increasing *x*, and the average grain size increases. The study of the dielectric constant and the tangent of the dielectric losses showed that the materials La_0.67_Li_*x*_Ti_1-*x*_Al_*x*_O_3_ have high values of the dielectric constant ε′ > 10^4^ in the frequency range of 0.01 ≤ *f* ≤ 10^3^ Hz. The obtained materials can be used in miniaturized electronic systems to solve problems of miniaturization.

## Author contribution statement

Tetiana Plutenko: conceived and designed the experiments; performed the experiments; analyzed and interpreted the data; wrote the paper.

Oleg V’yunov; Oleksandr Fedorchuk: analyzed and interpreted the data; wrote the paper.

Borys Khomenko: contributed reagents, materials, analysis tools or data, performed the experiments.

Anatolii Belous: contributed reagents, materials, analysis tools or data; wrote the paper.

## Data availability statement

Data included in article/supp. material/referenced in article.

## Additional information

No additional information is available for this paper.
